# Stability Indicating RP-HPLC Estimation of Nebivolol Hydrochloride in Pharmaceutical Formulations

**DOI:** 10.4103/0250-474X.45396

**Published:** 2008

**Authors:** D. A. Shah, K. K. Bhatt, R. S. Mehta, S. L. Baldania, T. R. Gandhi

**Affiliations:** Anand Pharmacy College, Opp. Town Hall, Anand-388 001, India; 1 A. R. College of Pharmacy, P. Box No. 19, Vallabh Vidyanagar-388 120, India

**Keywords:** Nebivolol hydrochloride (NEB), degradation, reversed phase liquid chromatography, stability indicating, validation

## Abstract

A simple, specific, accurate and stability indicating reversed phase liquid chromatographic method was developed for the determination of nebivolol hydrochloride in tablet dosage forms. A phenomenex Gemini C-18, 5 μm column having 250×4.6 mm i.d., with mobile phase containing methanol: acetonitrile: 0.02 M potassium dihydrogen phosphate (60:30:10, v/v/v; pH 4.0) was used. The retention time of nebivolol hydrochloride was 2.6 min. The linearity for nebivolol hydrochloride was in the range of 0.2-10 μg/ml. The recovery was found to be in the range of 98.68-100.86%. The detection limit and quantification limit were found to be 0.06 μg/ml and 0.2 μg/ml, respectively. Nebivolol stock solutions were subjected to acid, alkali and neutral hydrolysis, chemical oxidation and dry heat degradation. The degraded product peaks were well resolved from the pure drug peak with significant difference in their retention time values. The proposed method was validated and successfully applied to the estimation of nebivolol hydrochloride in tablet formulations.

Beta-blockers are standard therapy in patients with cardiovascular disease, and are the preferred drugs in the treatment of both hypertension and chronic heart failure^1^. Nebivolol hydrochloride (NEB) is chemically α,α'-[Iminobis(methylene)]bis-[6-fluoro-3,4-dihydro-*2H*-1-benzopyran-2-methanol], hydrochloride. It is a third-generation, cardio-selective beta-blocker used in the treatment of mild to moderate essential hypertension and chronic heart failure^2^. The mode of action of NEB is distinctly different from atenolol and other beta-blockers. It acts by producing vasodilatation and improves endothelial function via the arginine-nitric oxide pathway^3^–^5^.

Literature survey revealed that methods have been reported for the estimation of NEB in plasma using liquid chromatography^6^ (LC) coupled with electro-spray ionization tandem mass spectrometry and for the enantiomeric resolution of drug using liquid chromatography^7^–^8^. The aim of present study was to develop a new, selective and sensitive liquid chromatographic method for the estimation of NEB in tablets for routine quality control analysis and stability studies.

## MATERIALS AND METHODS

The liquid chromatographic system of Shimadzu make containing LC-10AT (VP series) pump, Variable wavelength programmable UV/Vis detector SPD-10AVP and Rheodyne injector (7725i) with 20 μl fixed loop was used. Chromatographic analysis was performed using Spinchrom software. A Phenomenex Gemini C18 column with 250×4.6mm i.d. and 5 μm particle size was used. Analytically pure NEB was obtained as gift sample from M/s Torrent Pharmaceutical Ltd., (Ahmedabad, India). Methanol, acetonitrile, water (E. Merck, Mumbai, India) were of LC grade, while potassium dihydrogen phosphate (S. D. Fine Chemicals, Mumbai, India) was of analytical grade used for the preparation of mobile phase. Tablet formulation A (Nodon, Cadila Pharmaceuticals Ltd., India) and B (Nebilol, J. B. Chemicals Ltd., India) containing labeled amount of 5.45 mg of NEB were purchased from local market.

### Preparation of mobile phase and stock solution:

Potassium dihydrogen phosphate was weighed (0.272 g) and dissolved in 100 ml of water. The pH of the solution was adjusted to 4 with the help of 0.1 M phosphoric acid. The solution was mixed with 600 ml of methanol and 300 ml of acetonitrile. The solution was sonicated for 10 minutes and filtered using Whatman filter paper (No.1) and used as mobile phase. Stock solution was prepared by weighing (25 mg) and transferring analytically pure NEB to 25 ml volumetric flask. Volume was made up to the mark with methanol, which gave 1000 μg/ml of the drug. The solution was further diluted with the same solvent to obtain final concentration of 100 μg/ml.

### Chromatographic conditions:

A reverse phase C18 column equilibrated with mobile phase methanol: acetonitrile: 0.02 M potassium dihydrogen phosphate (60:30:10, v/v/v; pH 4.0) was used. Mobile phase flow rate was maintained at 1 ml/min and effluents were monitored at 280 nm. The sample was injected using a 20 μl fixed loop, and the total run time was 10 min.

### Calibration curve for NEB:

Appropriate aliquots of stock solution were taken in different 10 ml volumetric flasks and diluted up to the mark with mobile phase to obtain final concentrations of 0.2, 0.6, 1, 2, 4, 6, 8, 10 μg/ml of NEB, respectively. The solutions were injected using a 20 μl fixed loop system and chromatograms were recorded. Calibration curve was constructed by plotting average peak area versus concentrations and regression equation was computed for NEB.

### Determination of NEB in pharmaceutical formulations:

Twenty tablets were weighed and finely powdered. Powder equivalent to 10 mg NEB was accurately weighed and transferred to a 50 ml volumetric flask and 20 ml of methanol was added to the same. The flask was sonnicated for 20 min and volume was made up to the mark with methanol. The above solution was filtered using Whatman filter paper (No.1). Appropriate volume of the aliquot was transferred to a 25 ml volumetric flask and the volume was made up to the mark with mobile phase to obtain 4 μg/ml of NEB. The solution was sonicated for 10 min and injected under above chromatographic conditions and peak area was measured.

### Validation:

The calibration curve was obtained at 8 concentration levels of NEB standard solutions. The solutions (20 μl) were injected into liquid chromatographic system (n=5) with chromatographic conditions previously given. The linearity was evaluated by least square regression method. The accuracy of the method was determined by calculating recoveries of NEB by method of standard additions. Known amount of drug (0, 0.4, 4, 8 μg/ml) were added to a pre-quantified sample solution and the amounts were estimated.

The intra-day and inter-day precision study was carried out by estimating the corresponding responses 3 times on the same day and on 3 different days (first, second and third day) for 3 different concentrations of NEB (0.6, 4, 10 μg/ ml), which represents low, medium and high concentrations in the analytical range. The specificity was estimated by spiking commonly used excipient (starch, talc and magnesium stearate) into a pre weighed quantity of drug. The chromatogram was taken by appropriate dilutions and the quantities of drugs were determined.

The detection limit is defined as the lowest concentration of an analyte that can reliably be differentiated from background levels. Limit of quantification of an individual analytical procedure is the lowest amount of analyte that can be quantitatively determined with suitable precision and accuracy. LOD was the concentration that yielded signal to noise ratio (S/N) 3:1 and LOQ was the concentration that yielded signal to noise ratio (S/N) 10:1.

Robustness of the method was studied by changing the composition of organic phase by ± 5% and also by observing the stability of the sample solution at room temperature for 24 h in dark. The sample solution was assayed at every 6 h interval up to 24 h.

### Forced degradation study:

Analytically pure NEB was weighed (25 mg each) and transferred to 25 ml volumetric flask and diluted up to the mark with methanol. This stock solution was used for forced degradation study. To study forced degradation by hydrolysis, 1 ml of stock solution was taken in different 10 ml volumetric flask and boiled for 2 h at 80° after adding 5 ml of 0.1 N HCl for acid hydrolysis, 5 ml of 0.1 N NaOH for base hydrolysis, 5 ml of water for neutral hydrolysis, individually. To perform chemical oxidation, 1 ml of stock solution was taken in 10 ml volumetric flask and 5 ml of 3% hydrogen peroxide was added. The solution was boiled for 2 h at 80°. Dry heat degradation study of the drug was carried out by exposing bulk drug in oven at 90° for 2 h. After heating, 25 mg of drug was weighed and transferred to 25 ml volumetric flask and volume was made up to the mark with methanol. Each stressed sample was diluted with mobile phase to obtain final concentration of 5 μg/ml for NEB and each solution was analyzed in triplicate.

## RESULTS AND DISCUSSION

Optimization of mobile phase was carried out by taking different proportions of aqueous and organic phases to obtain rapid, simple assay method for NEB with appropriate run time, asymmetric factor and theoretical plates. Mobile phase consisting of methanol: acetonitrile: 0.02 M potassium dihydrogen phosphate (60:30:10, v/v/v; pH 4.0) was found to be satisfactory which gave symmetric and sharp peak for NEB at a 1 ml/min flow rate. The UV spectrum of the drug shows absorption bands at 203, 216 and 280 nm. For quantitative analytical purpose wavelength was set at 280 nm, which provided better reproducibility with minimum interference than the other UV bands. Under the chosen experimental conditions, the liquid chromatogram of NEB showed a single peak of the drug around retention time (Rt) 2.6 min with asymmetry 1.4.

The calibration curve for NEB was obtained by plotting the peak area versus concentration. It was found to be linear in the range of 0.2-10 μg/ml. Peak area and concentrations were subjected to least square regression analysis to calculate calibration equation and correlation coefficient. The data of the calibration curve are shown in Table 1. The correlation coefficient (r) was found to be 0.9991, showing good linearity. Accuracy of the method was examined by performing recovery studies by standard addition method. The recovery of the added standard to the sample was calculated and it was found to be 98.68–100.86%, which indicated good accuracy of the method. Precision studies were carried out at 3 different concentration levels (0.6, 4, 10 μg/ml) and the results of the intra-day and inter-day studies are reported in terms of RSD (Table 2). In specificity study a single peak was obtained for NEB with recoveries in the range of 98.32-100.79%, which indicate that there was no interference from the excipient used. The LOD value was found to be 0.06 μg/ml which is the concentration that yields signal to noise (S/N) ratio 3:1. The LOQ was 0.2 μg/ml with S/N ratio of 10:1. The RSD value of the assay of NEB during solution stability studies were with in 2%. The data proves that the sample solutions were stable up to 24 h at room temperature.

**TABLE 1:** REGRESSION ANALYSIS OF THE CALIBRATION CURVE FOR THE ASSAY OF NEB

Parameters	NEB
Linearity range (μg/ml)	0.2-10
Slope	34.00
Standard deviation of slope	0.315
Intercept	0.0027
Standard deviation of intercept	1.24
Correlation coefficient (r)	0.9991

**TABLE 2:** SUMMARY OF VALIDATION PARAMETERS FOR ASSAY OF NEB

Parameters	NEB
Detection limit (μg/ml)	0.06
Quantitation limit (μg/ml)	0.2
Accuracy(%)	98.68-100.86%
Precision (RSD^a^,%)	
Intra-day (n=3)	0.67-1.45
Inter-day (n=3)	0.91-1.85

a RSD indicates relative standard deviation.

The proposed liquid chromatographic method was applied to the determination of NEB in Tablet formulation A and B. The results obtained were satisfactorily accurate and precise as indicated by the good recovery and standard deviation (SD) values (Table 3).

**TABLE 3:** ASSAY RESULTS OF TABLET DOSAGE FORM USING PROPOSED METHOD

Formulations	Amount of drug taken (mg)^b^	Amount obtained (mg)^b^	% Recovery^b^
A	10	10.04±0.170	100.36±1.70
B	10	9.88±0.079	98.80±0.79

b mean value±standard deviation of three determinations; Tablet formulation A is Nodon (Cadila Pharmaceuticals Ltd., India) and B is Nebilol (J. B. Chemicals Ltd., India) containing labeled amount of 5.45 mg of NEB.

Forced degradation study was carried out as per ICH guideline^9^. The objective of the study was to find out the likely degradation products, which in turn help in the establishment of degradation pathways and the intrinsic stability of drug molecule. In order to check the selectivity of the proposed method, degradation studies were carried out using hydrolysis (acidic, basic, and neutral), thermolysis and chemical oxidation.

**Fig. 1:**
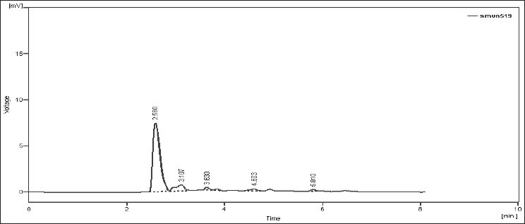
Chromatogram of base-treated NEB. Chromatogram of base (0.1 N NaOH) hydrolyzed nebivolol hydrochloride (NEB) at 80° for 2 h showing degradation peaks at retention time (Rt) 3.107 min, 3.630 min, 4.603 min, 5.810 min.

**Fig. 2:**
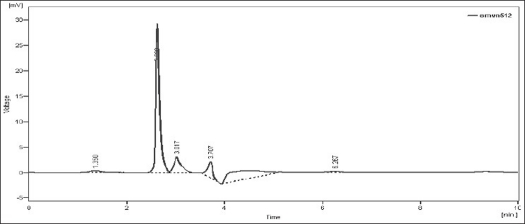
Chromatogram of acid-treated NEB. Chromatogram of acid (0.1 N HCl) hydrolyzed nebivolol hydrochloride (NEB) at 80° for 2 h showing degradation peaks at retention time (Rt) 3.017 min, 3.707 min, 6.267 min.

**Fig. 3:**
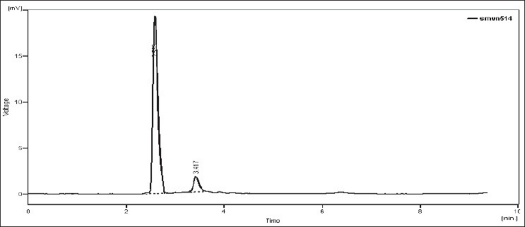
Chromatogram of H_2_O_2_-treated NEB. Chromatogram of chemically oxidized nebivolol hydrochloride (NEB) using 3% H_2_O_2_ showing degradation peak at Rt 3.417 min.

Upon heating the drug solution with 0.1 N NaOH for 2 h at 80°, a 20% reduction in the original drug peak was observed and it showed additional peaks at retention time (Rt) 3.107, 3.630, 4.603, 5.810. The peaks of the degraded products were well resolved from the drug peak (fig. 1). When NEB solution was exposed for acid hydrolysis, the chromatographic peak corresponding to the parent drug diminished about 11% and showed additional peaks at Rt 3.017, 3.707, 6.267 (fig. 2). When NEB was subjected to dry heat degradation study, one new signal was observed at Rt 3.080. The chromatogram of neutral hydrolysis study showed additional peak at Rt 2.973, which indicated that the drug was susceptible to hydrolysis by water at 80°. The degradation product peak was not properly resolved from the drug peak. When NEB was subjected to chemical oxidation using hydrogen peroxide, the additional peak was observed at Rt 3.417 (fig. 3). Forced degradation study under described condition showed that, NEB degraded partially with neutral hydrolysis, chemical oxidation and thermal stress conditions and, was very susceptible to acidic and basic hydrolysis (Table 4).

**TABLE 4:** DEGRADATION OF NEBIVOLOL HYDROCHLORIDE.

Condition	Time (h)	Recovery (%)	Retention time of degradation products (min)
Base 0.1 N NaOH^c^	2	80.75	3.107, 3.630, 4.603, 5.810
Acid 0.1 N HCl^c^	2	89.68	3.017, 3.707, 6.267
Wet heat^c^	2	97.85	2.973
Dry heat (90°)	2	98.42	3.080
3% H_2_O_2_	2	98.13	3.417

c Sample is heated at 80° for specified period of time.

In proposed study, stability indicating liquid chromatographic method has been developed for determination of NEB in tablet dosage form. The method was validated and found to be simple, sensitive, accurate and precise. Statistical analysis proved that method was repeatable and selective for the analysis of NEB without any interference from the excipient. The method was successfully used for determination of drugs in their pharmaceutical formulations. Also the above results indicate the suitability of the method for acid, base, thermal and wet heat degradation and chemical oxidation study. As the method separates the drug from its degradation products, it can be used for analysis of stability samples.
